# In silico fragmentation for computer assisted identification of metabolite mass spectra

**DOI:** 10.1186/1471-2105-11-148

**Published:** 2010-03-22

**Authors:** Sebastian Wolf, Stephan Schmidt, Matthias Müller-Hannemann, Steffen Neumann

**Affiliations:** 1 Leibniz Institute of Plant Biochemistry- Department of Stress- and Developmental Biology, Weinberg 3, 06120 Halle(Saale), Germany; 2 Institut für Informatik, Martin-Luther-Universität, Halle-Wittenberg, Von-Seckendorffplatz 1, 06120 Halle (Saale), Germany

## Abstract

**Background:**

Mass spectrometry has become the analytical method of choice in metabolomics research. The identification of unknown compounds is the main bottleneck. In addition to the precursor mass, tandem MS spectra carry informative fragment peaks, but the coverage of spectral libraries of measured reference compounds are far from covering the complete chemical space. Compound libraries such as PubChem or KEGG describe a larger number of compounds, which can be used to compare their in silico fragmentation with spectra of unknown metabolites.

**Results:**

We created the MetFrag suite to obtain a candidate list from compound libraries based on the precursor mass, subsequently ranked by the agreement between measured and in silico fragments. In the evaluation MetFrag was able to rank most of the correct compounds within the top 3 candidates returned by an exact mass query in KEGG. Compared to a previously published study, MetFrag obtained better results than the commercial MassFrontier software. Especially for large compound libraries, the candidates with a good score show a high structural similarity or just different stereochemistry, a subsequent clustering based on chemical distances reduces this redundancy. The in silico fragmentation requires less than a second to process a molecule, and MetFrag performs a search in KEGG or PubChem on average within 30 to 300 seconds, respectively, on an average desktop PC.

**Conclusions:**

We presented a method that is able to identify small molecules from tandem MS measurements, even without spectral reference data or a large set of fragmentation rules. With today's massive general purpose compound libraries we obtain dozens of very similar candidates, which still allows a confident estimate of the correct compound class. Our tool MetFrag improves the identification of unknown substances from tandem MS spectra and delivers better results than comparable commercial software. MetFrag is available through a web application, web services and as java library. The web frontend allows the end-user to analyse single spectra and browse the results, whereas the web service and console application are aimed to perform batch searches and evaluation.

## Background

Mass spectrometry has become the analytical method of choice in metabolomics research [1]. Various ionisation methods are commonly used, such as electron impact (EI) used with gas chromatography (GC/MS), or the soft electrospray ionisation (ESI), which is employed in LC/ESI-MS systems. The main bottleneck in the interpretation of metabolomics experiments is the identification of compounds. In addition to the exact mass, tandem MS spectra provide additional structural hints, providing a fingerprint of the measured molecule. In tandem MS, the molecules are interacting with a collision gas at specified kinetic energies, hence the name *collision induced dissociation*. Large spectral libraries of measured reference spectra exist for GC/MS, such as the commercial NIST library '08 (Gaithersburg, MD) or the GMD [2], but for ESI-tandem MS spectral libraries are still few and comparably small [3,4]. A different approach towards identification is the interpretation of the measured spectra, usually with regard to the known (or hypothetical) molecular structure.

*Fragmenter with a rule set *like the commercial tools ACD Fragmenter [5] and Mass Frontier [6] generate fragments based on cleavage rules known from the literature, in both cases the algorithmic details are not published. For some compounds, MassFrontier 5 is not able to identify any fragments in negative mode [7]. Hill et al. used Mass Frontier 4 to predict the tandem MS spectra of 102 test compounds, which were analysed using a Micromass Q-TOF II in positive mode, to identify the measured compound and its structure. Candidate compounds were retrieved from PubChem using the exact mass. MassFrontier used those structures as input and generated spectra which were compared to the measured spectra. Finally, the compounds were ranked according to the peaks common to both the predicted and measured spectra [8]. *Combinatorial Fragmenter *such as Fragment Identificator (FiD) proposed by Heinonen et al. [9] try to predict the fragmentation tree given both a metabolite's molecular structure and its tandem mass spectrum. Due to high computational complexity, even for a single medium sized compound (around 300 Da) runtimes can reach several hours. Another approach is the systematic bond disconnection method without a rule set as described in [10]. The resulting product ions from a single precursor structure are matched against the peaks measured with a high-resolution mass spectrometer. The software EPIC was tested against two hand annotated spectra from the literature and is not publicly available. The runtime was reported to be around 1 minute to process 1-(3-(5-(1,2,4-triazol-4-yl)-1H-indol-3-yl)propyl)-4-(2-(3-fluorophenyl)ethyl)piperazine (432 Da).

MetFrag is a combinatorial fragmenter using the bond disconnection approach, which is fast enough to screen dozens to thousands of candidates retrieved from e.g. KEGG, PubChem or ChemSpider compound databases. We do not attempt to create a mechanistically correct prediction of the fragmentation processes. Instead, we want to perform a search in compound libraries using the measured fragments as additional structural hints.

The paper is structured as follows: in the next section we describe the architecture and the in silico fragmentation algorithm, including heuristics to speed up calculations and to account for molecular rearrangements upon fragmentation. Afterwards, we explain the scoring function. In the results section we evaluate MetFrag on a set of 710 spectra from 151 compounds. The paper finishes with our conclusions. All detailed results are available as additional files.

## Implementation

**Figure 1:**
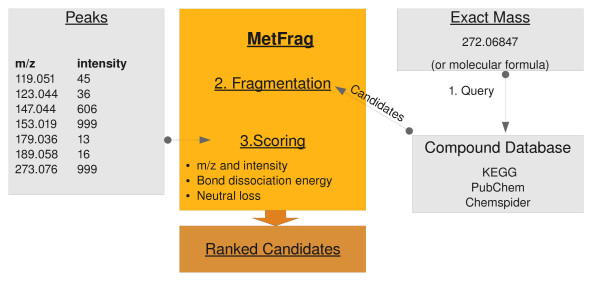
**Workflow of a search based on exact mass and tandem MS spectrum**. First the upstream compound library is searched using their respective web service API. The scoring ranks the measured peaks against the in silico fragments.

The workflow implemented in MetFrag is shown in Figure 1, and covered in detail in the following sections. MetFrag is implemented in Java and uses the Chemistry Development Kit [11], an open source Java library. The CDK provides algorithms and data structures for structural Chemo- and Bioinformatics and is able to read and write common formats such as MDL, CML, InChI, and many more.

### Retrieval of candidates from compound libraries

First we perform a search in a general purpose compound database for candidate molecules based on the exact mass (within an error range given in ppm) of the neutral and intact molecule. Currently three compound databases can be queried: KEGG Compound (about 16 021 entries, October 2009) [12], PubChem (37 million, June 2009) [13] and ChemSpider (23 million, October 2009) [14]. Optionally, the search can be restricted to compounds containing only the elements CHNOPS, commonly occurring in natural products.

Alternatively, the compound databases can be searched with the elemental composition if this has been derived from e.g. exact mass and isotopic pattern of the precursor. Finally, the set of candidates can be supplied by simply enumerating all database IDs to be processed, e.g. obtained by an independent search for metabolites of a pathway. To query other (local) libraries, a custom wrapper can be added which contains the search logic.

The results usually contain dozens to thousands of hits with a similar (or identical in case of isomeric compounds) mass. The databases are accessed via their webservice interface and the resulting candidate compounds are downloaded automatically. Hydrogens are added explicitly to the structure where necessary.

### In silico fragmentation of candidates

**Figure 2:**
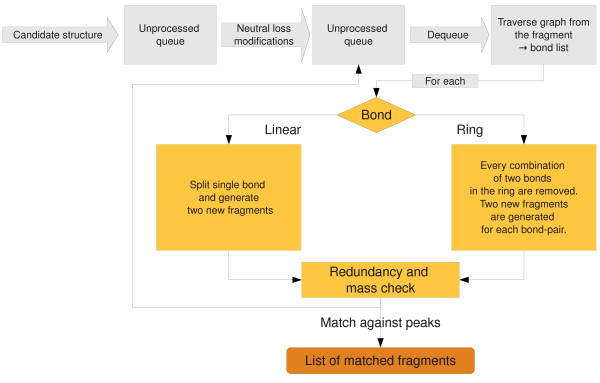
**Algorithm for in silico fragmentation**. Each compound is fragmented using the bond dissociation approach. Bonds in ring systems need special treatment. Every possible structure is generated until a given tree depth is reached. The redundancy heuristics and mass checks reduce the search space.

MetFrag generates all possible topological fragments of a candidate compound in order to match the fragment mass with the measured peaks. The problem of enumerating all possible molecular fragments can be solved by creating a fragmentation tree. The root consists of the intact molecule, and each node represents a fragment, obtained by splitting the molecule at a given bond. We implemented this as an iterative, breadth-first algorithm. One major speed determining factor is the number of fragments generated, because of the combinatorial nature of the algorithm. Thus, the *maximum tree depth *was introduced to improve the performance and specificity. We perform additional application-specific steps to prune the search space and take care of molecular rearrangements, see below. For each candidate structure the fragments are generated in the following way (Figure 2):

Initially the candidate structure is pushed into an "unprocessed" queue. The candidate structure is preprocessed using a (small) set of rules, which describe molecular rearrangements during the CID fragmentation that can not be accounted for by the simple bond disconnection approach. Each application of these rules results in one or more derived fragments which are added to the "unprocessed" queue. The actual rules will be described later in this paper.

Then a structure is dequeued and its molecular graph is traversed to collect all bonds to be split. A linear bond (which is not part of a ring system) only needs to be cleaved and results in two new fragments. Within a ring system two bonds have to be split simultaneously, to create the new fragments. Only the fragments larger than the peak with the smallest mass are created, since smaller fragments can not explain an experimental peak.

Before proceeding to the next fragment, a redundancy check is performed to eliminate duplicate fragments. Redundancy occurs if a fragment *A *is part of both parent fragments *AB *and *ABC*, or the fragment *A *appears in different places of the molecule, as in *ABA*. In both cases the redundant structures would cause longer runtimes and higher memory consumption without gaining any information. In addition to full (and time consuming) graph isomorphism checks we describe simpler heuristics later in this paper.

Finally, the in silico fragments are matched against the query peaklist. The measured peaks correspond to the charged fragments, so the matching function adds (positive mode) or removes (negative mode) a proton (1.007 Da) to the fragment mass. In a few cases, fragment ions can have an intrinsic charge, where one of the heteroatoms is charged. In this case the fragment mass is used as-is, but a penalty is added to the bond dissociation energy of this fragment (see below).

The accuracy of a mass measured by an MS instrument is typically expressed relatively in ppm. In practice we found that especially for low masses, an additional (absolute) deviation has to be considered. Hence MetFrag uses two values mzppm and mzabs respectively, to calculate the mass error used for fragment matching.

Peaks that have such an explanation are subsequently removed from the query peaklist and the fragment-peak pair is saved for the final scoring. If the peak with the smallest mass has been explained, this will raise the minimal-mass cut-off, resulting in even fewer fragments that need to be considered. The "unprocessed" queue is then populated with the created and filtered fragments and processed as described above. The fragmentation terminates if the queue is empty or the maximum tree depth has been reached. The candidate is then scored based on all matched fragment-peak pairs as explained in the following section.

### Scoring candidates based on fragments explaining the measured peaks

The score is an extension of a simple peak count: *S*_*i*_ of a candidate compound *i *is calculated based on all fragments *F*_*i *_that explain peaks in the measured spectrum and the bond dissociation energy (BDE) calculated during the in silico fragmentation:(1)

where

In general a peak with a high mass and intensity is more characteristic than peaks with lower mass and intensity. This is reflected by the weighted peak count *w*_*i*_, as already proposed by [3,15]. The exponents *m *= 0.6 and *n *= 3 we use are taken from the literature [15]. The weights *w*_*i *_are scaled by max(*w*) such that it is between 0 and 1. We also take the bond dissociation energy (BDE) into account, the higher the BDE, the less likely we consider a fragment. We use the standard enthalpy change upon bond fragmentation from literature, see e.g. [16]. For each candidate *f *we sum up BDE_*b *_for all bonds *B*_*f*_ cleaved along the fragmentation tree for the explained fragments *F_i_*. Afterwards, for each candidate the arithmetic mean *e*_*i *_of these BDEs is scaled by 2 max(*e*) such that it is between 0 and 0.5.

### Neutral loss rules account for rearrangements

The ionised molecules typically have a single charge. After the fragmentation, the charge remains with either of the resulting fragments, the other is neutral. Because only charged ions can be measured, the mass difference between the two charged ions before and after the fragmentation is referred to as the "neutral loss" [17].

**Figure 3:**
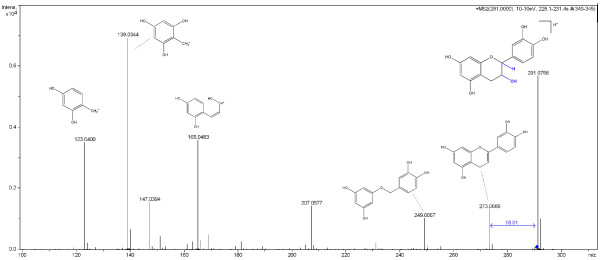
**Annotated tandem MS spectrum of Epicatechin**. This spectrum for Epicatechin was measured on a Bruker-micrOTOFQ mass spectrometer and manually annotated by an expert. The measured peaks and corresponding fragments for the major signals are depicted. In addition, the non-topological water loss is highlighted in blue.

One example of a common neutral loss is H_2_O, which is not a true substructure of any molecule. Instead, H_2_O is formed after a hydroxyl group (OH) and a single H are split off at *different *(though usually nearby) positions (see Figure 3, where the distance is three). Because individual H atoms are not considered during the in silico fragmentation, the resulting fragment would never be found without special treatment. MetFrag is checking for structural patterns that can lead to such a non-topological fragmentation. We check within a specified topological distance of the OH-group for another hydrogen and remove both OH and H.

This non-topological fragmentation is handled by the rules shown in Table 1, other neutral losses are covered by the bond-disconnection approach. Rules can be added easily, e.g. if the compounds measured belong to unusual compound classes. MetFrag reads these during start up and applies the rules to the initial candidates, resulting in new (derived) candidate molecules.

**Table 1:** Neutral loss rules

Ion Mode^***a***^	Exact Mass^***b***^	Topological Fragment^***c***^	Neutral Loss^***d***^	Maximum Distance^***e***^
+ -	18.0106	OH	H2O	3
+ -	27.0109	CN	HCN	3
+ -	17.0266	NH2	NH3	3
+ -	30.0106	COH	CH2O	3
+	46.0055	COOH	HCOOH	3

These rules are applied to the initial candidate structures to account for rearrangements during the tandem MS fragmentation, i.e. neutral losses of unconnected fragments: ^*a*^ionisation mode where this rule can be applied, ^*b*^exact mass in Da of the neutral loss, ^*c*^molecular formula of the characteristic fragment, ^*d*^all atoms that are removed, e maximum number of bonds traversed to match neutral loss.

### Elimination of redundant fragments

We implemented three alternative *structure redundancy checks*. Intuitively, a proper graph isomorphism check is the best approach to eliminate structures with the same molecular connectivity. In practice, graph isomorphism checks are not fast enough to process thousands of structures in reasonable time.

Alternatively we implemented an *atom based *redundancy check: each atom is labelled with a unique identifier and resulting fragments are compared to others based on atom IDs. This method will not detect the redundancy as in *ABA *mentioned above, because the atoms in the two identical substructures *A *carry different IDs. This method showed the same identification rate at much lower runtime requirements. To reduce the complexity of the test even further, the *molecular formula *redundancy check was introduced, which compares fragments based only on their elemental composition. This check will detect the *ABA *redundancy, but will produce false positives if two structures have the same elemental composition, but with different bond structure, i.e. connectivity. If two fragments have the same molecular formula, the one that requires the lower bond dissociation energy is chosen. This way the fragments which are more likely to occur are considered. The molecular formula redundancy check is used by default, because the results are comparable at considerably reduced runtime.

### Structure clustering

Depending on the upstream database, the MetFrag result list can contain many similar structures or stereo isomers which have identical MetFrag scores. Therefore, we cluster the hits with tied ranks using the pairwise Tanimoto [18] distance of the molecular fingerprints, as implemented in the CDK [11]. All hits with a pairwise similarity ≥ 0.95 are collapsed into one cluster.

### User interface and available APIs

**Figure 4:**
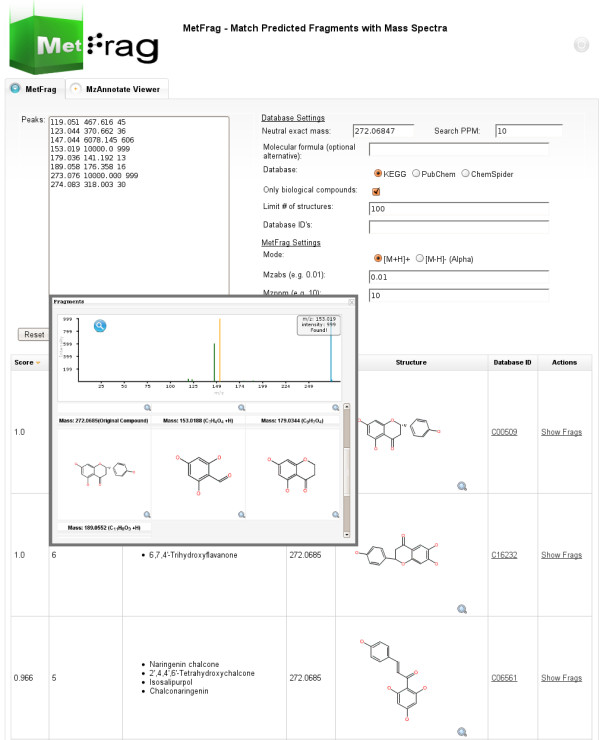
**MetFrag web interface**. The web interface with the search parameters at the top and the result list below. The extra window can be opened for each result and shows details such as the spectrum and matching fragment structures.

Our MetFrag application features an user friendly web interface, http://msbi.ipb-halle.de/MetFrag/. The required input includes the tandem MS peaklist with intensities (Figure 4, top left), selection of the upstream compound database and respective search parameters (top right). Alternatively, a list of database IDs can be provided explicitly. This allows e.g. to select the candidates based on their occurrence in specific pathways. Figure 4 also shows the results browser. A feedback form allows to store all input data, user rating of the hypotheses, and further comments. This helps to collect user-provided test- and training data. Spectra will *not *be saved unless explicitly granted. The web interface is based on Java Server Faces (JSF) [19], using the Apache MyFaces [20] implementation, ICEfaces [21] (a component library with AJAX capabilities) in an Apache Tomcat [22] servlet container. Thus, MetFrag is platform independent and accessible using most javascript enabled browsers.

We also provide a BioMoby [23] web service, which can be called from other software, including the Taverna workflow engine. Finally, the actual MetFrag algorithms are available as Java library, which can be used to perform batch searches and evaluation.

## Results and Discussion

In this section we give an example of MetFrag results for an exemplary compound, and describe the full test data sets and evaluation criteria. We evaluate MetFrag on two data sets, measured on different instruments, using either KEGG or PubChem as compound library.

For the evaluation we use the merged spectra from different collision energies of compounds where the database id is known. If MetFrag returns multiple hypotheses with tied ranks, we report the most pessimistic position: even if the correct solution has the highest observed score, if 9 other candidates also have the same score, then we assign rank 10.

In addition to the worst case rank we report the *cluster rank*. Clusters of compounds having a structural Tanimoto similarity ≥ 0.95 are collapsed and treated as one *compound cluster*. Again, this measure is quite conservative, because ranks are collapsed only within results having identical scores, and still the worst case cluster rank is reported. The standard deviation of both the raw and cluster ranks for a larger benchmark data set can be quite high, therefore we report not only the average rank, but also the median and 75% quantile.

### Example: Spectrum of Naringenin

As an example we show the analysis of a tandem MS spectrum of Naringenin (C_15_H_12_O_5_, KEGG C00509) with MetFrag. Using KEGG as compound library with a realistic 10 ppm window around the exact mass of 272.068 Da will return 15 hits. Each candidate structure is retrieved and fragmented as described in the previous section.

**Figure 5:**
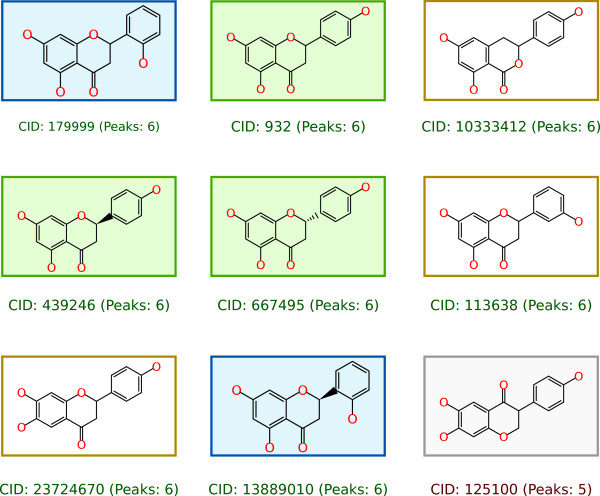
**Top candidates for Naringenin against PubChem**. The 9 top ranked compounds where the correct solution (CID 932) is reported at (tied) rank 8. Two clusters of structures (green and blue) are identical apart from their stereochemistry, the remaining three structures (yellow) that explain all six tandem MS peaks have a Tanimoto similarity < 0.95. After clustering with a similarity ≥ 0.95 the stereoisomers are collapsed into one cluster, resulting in a cluster rank 5 for the correct solution.

After scoring each structure, the first three results can be seen in Figure 4. The details window shows the fragments that can be explained by the spectrum. The same query in PubChem yields 736 candidates, and Figure 5 shows the 9 top ranked solutions, including the correct compound at worst case rank 8. The similarity would collapse the isomers into two clusters, resulting in a cluster rank 5.

### Benchmark data sets

Two data sets were used for evaluation, together consisting of 710 spectra of 151 known compounds. Current instruments allow the acquisition of so called *ramp *spectra, which combine a range of collision energies in one measurement. In both data sets the compounds were measured at different collision energies. Depending on the compound, informative fragmentation might occur only at higher energies. For other compounds, even low collision energies can lead to a very high degree of fragmentation. For this reason we use *composite *spectra: two peaks *p*_1 _and *p*_2 _from different collision energies are merged  = avg(mz_1_, mz_2_) if |mz_1 _- mz_2_| ≤ 0.01 Th, retaining the higher intensity max(*int*_1_, *int*_2_).

#### Data set I with compound library KEGG

The first data set consists of 200 spectra from 49 compounds obtained on the API QSTAR Pulsar I in positive mode at several different collision energies, e.g. 10, 20, 30 and 40 eV. The spectra were measured at the IPB and are publicly available in the MassBank database http://msbi.ipb-halle.de/MassBank/, see additional file 1 for a list of accession numbers.

MetFrag was used to identify the compounds using the 49 composite spectra within KEGG. Fragments are generated until a tree depth of two is reached. The instrument specific deviation was set to mzabs = 0.01 and mzppm = 50.

The initial list of candidates obtained from KEGG contained on average 10.3 compounds. The correct compound has a median of 3 in the MetFrag result list. 25 of the correct compounds were ranked in the top 3 hits and 11 of these are ranked first. MetFrag is a great improvement over a mass-only library search. With 16 021 entries KEGG is a comparably small library. However, the compounds are highly relevant to metabolomics research.

#### Data set II searched against PubChem

For the second data set we used the PubChem database, with a much larger collection of natural and synthetic compounds. A collection of 102 compounds with an average mass of 372.5 Da has been measured on a Micromass Q-TOF II in positive mode and published by Hill et al. in [8]. Each compound was measured at five different collision energies: 10, 20, 30, 40 and 50 eV, for an overall of 510 spectra. All spectra are available from MassBank as well, see additional file 2 for a list of accession numbers. For the spectra from this instrument we used 10 ppm (mzabs = 0) as mass deviation and a maximum tree depth of two. Based on a PubChem snapshot (June 2009) we retrieved on average 2508 candidate compounds.

After the MetFrag scoring, the correct candidate occurred at median rank 31.5, with the structure clustering the median decreased to 14.5. The complete results are shown in additional file 2.

We were also interested in the effect of a larger tree depth: raising the tree depth to three increases the average runtime 5-fold, and worse, the prediction accuracy decreases. The median of the correct compound degraded to 39 (cluster rank 18). This behaviour can be explained with the positive predictive value (PPV):

where

The more (smaller) fragments are generated, the more peaks can be matched, which leads to more false positive hits. This dependency is the reason to include the exponent  in the scoring function. The higher number of false positives results in a PPV of only 0.017 (tree depth three) versus 0.028 using tree depth of two.

Similarly, we applied the neutral loss rules (Table 1) to *every *generated fragment, not just the initial candidates. Again, we obtained more matching fragments, and the PPV decreased from 0.028 to 0.017, with an even higher median of the correct compound cluster of 67.

Another aspect of the evaluation was to use individual spectra instead of the composite spectra. MetFrag showed a poor performance resulting in a median of 43 using 10 ppm. An interesting observation is that the median improved to 39.5 if the allowed mass deviation is increased from 10 ppm to 20 ppm. Because the merging (and averaging) of peaks in the composite spectra usually results in a more accurate mass, some peaks in individual spectra with a deviation beyond 10 ppm are only matched after relaxing the allowed error window to 20 ppm.

Finally, we interpreted some of the cases where MetFrag did not return good results. Table 2 shows many top 10 hits, but also several cases where MetFrag is not able to rank the correct compound even among the top 100. Some of the problematic compounds are Ormetoprim, Strychnine N-oxide and Tetramisole. One reason is the high number of very similar candidate structures, and the difficulty to distinguish them based on the predicted spectra. Another example where many similar structures occur is Tetracycline, but here the rather high rank decreased from 92 to cluster rank 10. Even these large result lists with many similar entries will still give a very good estimation of the possible compound class, which simplifies the subsequent (manual) interpretation and identification.

**Table 2:** Results for data set II searched against PubChem

Compound	Candidates	MassFrontier Rank	Candidates	MetFrag Rank	Cluster Rank
Thioridazine	849	1	1091	1	1
Bumetanide	619	10	768	1	1
Piperacetazine	494	1	626	1	1
Sufentanil	445	1	512	1	1
Diphenoxylate	333	4	369	1	1
Tetracaine	308	22	362	1	1
Remifentanil	246	1	286	1	1
Hydroxybutorphanol	180	2	201	1	1
Alfentanil	134	1	162	1	1
Etamiphylline	100	3	104	1	1
Ergoloid Mesylate	7	1	10	1	1
Gallamine	10	1	8	1	1
Thonzide	4	1	4	1	1
Spectinomycin	310	1	361	2	1
Methionine Enkephalin	66	1	68	2	1
Leucine Enkephalin	53	2	60	2	1
Dihydroergotamine	35	1	38	2	1
Thiothixene	726	1	909	3	1
Etodolac	420	1	580	3	1
Prednisolone Tebutate	143	4	165	3	1
Oxybutynin	114	6	156	3	1
Apramycin	54	1	60	3	1
Tenoxicam	28	1	34	3	1
Vecuronium	3	1	4	3	1
Methylergonovine	515	1	629	6	1
Rolitetracycline	105	1	151	6	1
Oxytetracycline	483	4	614	11	1
Tetracycline	529	5	673	19	1
Thiethylperazine	569	2	671	2	2
Acetophenazine	435	1	546	2	2
Mebeverine	96	2	112	2	2
Salmeterol	32	1	37	2	2
Terfenadine	34	1	35	2	2
Boldenone Undecylenate	21	2	32	2	2
Buspirone	36	1	31	2	2
Gingerol	182	2	195	3	2
Betaxolol	190	5	259	4	2
Fenoterol	370	5	521	6	2
Taurocholate	59	4	65	9	2
Aminophylline	94	21	176	3	3
Sulfadimethoxine	94	18	145	3	3
Adiphenine	623	6	796	4	3
Perindopril	102	2	119	6	3
Sulfasalazine	106	5	116	6	3
Anileridine	563	251	668	7	3
Prednisolone	269	13	363	8	3
Adenosine Diphosphate	32	3	46	9	3
⋮	⋮	⋮	⋮	⋮	⋮
					
Tetramisole	120	1	123	85	79
Oxaprozin	461	101	607	143	94
Antipyrine	306	97	341	122	104
Mefenamic Acid	579	328	633	146	124
Strychnine	664	575	882	259	171
Dimefline	644	644	876	294	175
Ormetoprim	270	124	317	233	191
Strychnine N-oxide	1185	1098	1672	1012	618

**Average:**	**272.2 (± 24.2)**	**44.2 (± 14.1)**	**338.4 (± 31.5)**	**34.2 (± 10.9)**	**21.6 (± 6.8)**
**Median:**	**183.5**	**4**	**231.5**	**8**	**4**
**75% Quantile:**	**431.3**	**17.5**	**518.8**	**19**	**11.8**
**Std. Deviation:**	**244.1**	**142.4**	**318.1**	**109.8**	**69.1**

The results on the left were reported in [8]. The corresponding MetFrag results are on the right where the candidate search was restricted to the PubChem as of February 2006 (we retrieved slightly more candidates than reported by Hill et. al.). Only the best 47 and eight worst Metfrag results are shown, the full table is given as additional file 3.

We also evaluated data set I (measured on the API QSTAR Pulsar I) against PubChem 2009. Because this older mass spectrometer has a much lower mass accuracy than the Micromass Q-TOF II, both the candidate search and the scoring found more false positive matches. Within the 3896 (average) candidates, the median of the correct solution is only 91. This leads to the conclusion that a good mass accuracy of ≤10 ppm is required. Almost all current QTOF instruments are specified at 5 ppm or less, and even higher accuracies are available from Orbitrap or FTICR-MS instruments.

#### Comparison between MetFrag and MassFrontier

In their paper [8] Hill et al. evaluate the prediction performance of MassFrontier 4.0 with an approach similar to MetFrag, using PubChem (in the version from February 2006, with 6·10^6 ^entries) as compound database. We added a constraint to our candidate search to include only compounds added in or before February 2006. Our simulated PubChem snapshot returns on average 338 candidates, the previous study only 272 structures. Nevertheless, we use following results to compare MetFrag and MassFrontier. Both MetFrag and the search procedure by Hill consider only compounds containing the elements CHNOPS and ignore molecules which consist of C, H only. The previous study reports two separate evaluation strategies: the first combines the automatic ranking with the manual a-posteriori selection of the best spectrum, obtaining the correct result on a median rank 2.5. In practice, this knowledge will not be readily available. The more realistic results are presented in the supplementary material of [8], where a heuristic was used to select one spectrum per compound. The heuristic rule chooses the spectrum with the lowest collision energy which has at most 22% of the precursor ion intensity. In this case the median drops to 4 (3^*rd *^quantile at 17.5).

The median for MetFrag is 8 (3^*rd *^quantile at 19), and decreases to 4 (3^*rd *^quantile at 11.75) if the 95% similarity criterion is used. If the results are compared in more detail, this improvement is significant (*p *= 0.01), tested with a one-tailed, paired Wilcoxon signed rank test. The results for both systems are available as additional file 3.

It would be interesting to evaluate the MassFrontier approach with composite or ramp spectra, where neither automatic nor manual spectra selection would be required.

### Empirical runtime evaluation

**Figure 6:**
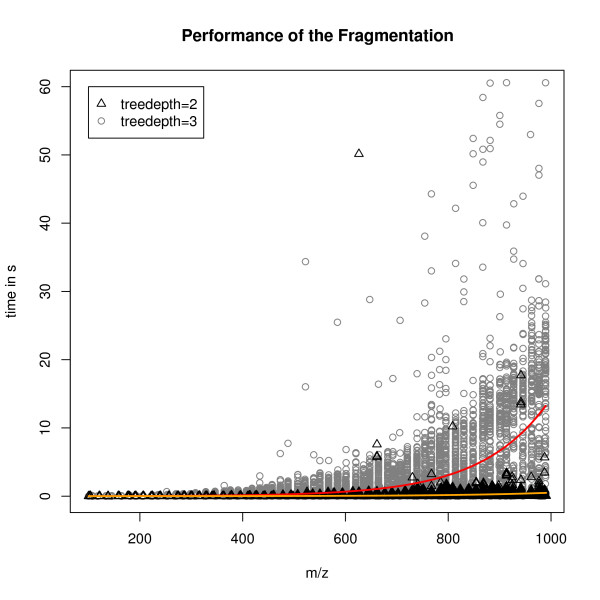
**Empirical runtime**. Runtime for the in silico fragmentation step on 5900 compounds randomly drawn from PubChem, with uniform mass distribution between 100 and 1000 Da. Limiting the tree depth of the in silico fragmentation to two (orange) results in an average runtime of 0.2 s for one compound. The exponential runtime can be seen especially when a larger tree depth (red) is used, raising the runtime to 3.4s.

The naïve and recursive bond-disconnection approach has very high theoretical complexity. We evaluated the real-world runtime by sampling 5900 compounds (unrelated to the test sets) from PubChem with a mass between 100 and 1000 Da. In metabolomics research, only few compounds exceed a mass of 1000. Each compound was fragmented (minimum fragment mass 30 Da) to a given tree depth of two and three. Figure 6 shows the runtime of MetFrag on a PC with Intel Q9400 CPU at 2.66 Ghz and 8 Gb RAM with Ubuntu 8.04, and JVM Sun Java 1.6.0_16-b01. Each point shows the time needed to compute all fragments above 30 Da. The yellow and red lines show the non-linear runtime for tree depth two (on average 0.2 s) or three (on average 3.4s), respectively. In practice a tree depth of two has the best prediction accuracy (see above) and is fast enough to analyse compounds on demand, even with masses up to 1000 Da.

## Conclusions

We have presented an algorithm which is able to identify small molecules from tandem MS measurements among a large set of candidate structures. The scoring function does not require a set of fragmentation reactions or an actual simulation of the fragmentation process. MetFrag is able to query KEGG, PubChem and ChemSpider, and local databases can be integrated with little effort.

In comparison to the system described in [8] (which included human expertise), MetFrag achieves better results than MassFrontier.

For dedicated metabolite databases such as KEGG, the correct identification is generally among the first few candidates. Given the sheer size of generic compound libraries such as PubChem, it is no surprise that the result lists contain many structurally highly similar compounds. Hence, an unambiguous identification is generally not possible, but usually the compound class can be derived from the results. A principal limitation is the inability to distinguish stereoisomers which is not possible from MS data alone. The final identification according to MSI recommendations [24] requires the comparison against spectra of authentic standards, or even complementary analysis methods such as NMR.

Our tool MetFrag improves the identification of unknown substances from tandem MS spectra. It is fast enough to be used in the interactive web application, and has a user-friendly interface and result browser.

## Availability and Requirements

• Project home page: http://metware.org/

• Operating system(s): Platform independent

• Programming language: Java

• Other requirements: Java ≥ 1.6, Tomcat ≥ 6.0

• License: GNU LGPL v3 (or later)

## Authors' contributions

SW implemented the MetFrag application, web interface and performed the evaluation. SS provided the MS expertise, MM-H and SN provided input for the requirements, the algorithmic design and architecture. All authors contributed to, read and approved the final manuscript.

## Supplementary Material

Additional file 1 **MassBank_KEGG_results**. Full list of mass spectra and compounds used in section "Data set I searched against KEGG". This includes accession numbers in the MassBank system. For each compound the number of candidates and the rank of the correct solution is given. Click here for file

Additional file 2 **HillData_PubChem2009**. Full list of mass spectra and compounds used in section "Data set II searched against PubChem". This includes accession numbers in the MassBank system. For each compound the number of candidates and the rank of the correct solution is given. Click here for file

Additional file 3 **Comparison_MassFrontier_MetFrag_PubChem2006**. This file includes the full results from table 2 in section "Data set II searched against PubChem". The candidate search was restricted to the PubChem as of February 2006. For convenience, we also include the results reported in [8]. Click here for file
